# The Impact of Workplace Bullying on Turnover Intention and Psychological Distress: The Indirect Role of Support from Supervisors

**DOI:** 10.3390/ijerph21060751

**Published:** 2024-06-08

**Authors:** Pascal Malola, Pascale Desrumaux, Eric Dose, Christine Jeoffrion

**Affiliations:** 1 Laboratory of Psychology of Pays de la Loire (LPPL–EA 4638), University of Nantes, 44000 Nantes, France; 2 ULR 4072—PSITEC—Psychologie: Interactions, Temps, Emotions, Cognitions, University of Lille, 59000 Lille, France; pascale.desrumaux@univ-lille.fr (P.D.); eric.dose@outlook.com (E.D.); 3 Université Grenoble Alpes, Université Savoie Mont Blanc, LIP/PC2S, 38000 Grenoble, France; christine.jeoffrion@univ-grenoble-alpes.fr

**Keywords:** workplace bullying, turnover intention, psychological distress, supervisor support

## Abstract

Workplace bullying is characterized by negative, repetitive, and frequent behaviors towards a person, affecting his/her physical and mental health The present study aimed to assess the relationship between bullying, turnover intention, and psychological distress, considering the potential mediating effect of perceived supervisor support. A questionnaire was completed by 252 women and 172 men (*n* = 424) from 70 French companies and institutions. They were working in private (70%), public (28%), and parapublic (2%) sectors. Finally, 33 trades are represented in this study: commercial (21%), educational (12%), medical (8.3%), and industry (8.3%) were the most prominently represented. Regression analyses showed that bullying was significantly linked to turnover intention (*ß* = 0.52, *p* < 0.05) and psychological distress (*ß* = 0.78, *p* < 0.001). Moreover, supervisor support played a mediating role between workplace bullying and turnover intention, as well as between workplace bullying and psychological distress. The implications and perspectives of the present research were subsequently discussed.

## 1. Introduction

The world of work has undergone dramatic change in recent years. In addition to physical hardship, other forms of work-related suffering have attracted more attention. Workplace bullying is defined as the accumulation, over a relatively extended period (of at least 6 months), of hostile words and actions expressed or manifested by one or more individuals towards another party (the target) [1]. It is also identified as suffering which changes people’s relationship with their work [2,3,4,5]. Workplace bullying is a serious issue for both employers and employees. One of the striking paradoxes is that there is virtually no empirical evidence regarding the effectiveness of interventions to prevent or reduce workplace bullying [6]. Over the years, an extensive body of research has been devoted to this issue, leading to a better understanding of the negative effects of bullying in the workplace [7,8,9,10,11,12]. For example, in a literature review on workplace bullying, Trepanier and colleagues [13] identified four main categories of antecedents: job characteristics, quality of interpersonal relationships, leadership styles, and organizational culture. As consequences, the authors reported positive associations with professional exhaustion, psychological distress, and absenteeism, as well as negative associations with work motivation, professional engagement, and the intention to leave the company. The findings of these studies not only highlight the negative impacts of workplace bullying as correlates with individual factors among targets but, more importantly, they underscore its adverse effects on organizations. For example, a one-year prospective study [14] found that exposure to bullying was linked to professional exhaustion at the one-year follow-up. Similarly, in a longitudinal study over a six-month period, Nielsen and colleagues [15] observed that workplace bullying was a robust predictor of psychological distress, including an unfavorable safety climate, tyrannical leadership, and high professional demands. Additionally, a study involving over 2000 employees over a one-year period revealed that exposure to workplace bullying was a stronger and more significant predictor of psychological distress than work-related stress [16]. Furthermore, workplace bullying detrimentally affects the well-being of employees, as it diminishes professional engagement and motivation [14,17] and increases absenteeism, the intention to leave the company [12,18,19,20,21,22,23,24], and symptoms of professional exhaustion [19,25]. Subsequently, in a literature review, ref. [7] demonstrates that workplace bullying has a negative impact on targets (psychological distress, burnout, suicidal thoughts, insomnia, and stress) and on the organization (intention to leave the company, retirement due to disability, absenteeism due to illness, role ambiguity, and role confusion). More recently, in a study, ref. [26] shows that workplace bullying negatively affects the psychological health of witnesses (professional exhaustion, work engagement). These results have been corroborated by previous studies. For instance, authors have found that being a witness to bullying harms psychological health (work-related depression and anxiety) and increases insomnia, headaches, and fatigue [27,28].

Such outcomes incur significant costs for organizations, ranging from recruitment challenges to increased employee turnover intentions and absenteeism. For the targets, their health is profoundly impacted, which represents a substantial cost if the phenomenon is left unaddressed. Given the vast literature on the correlates of bullying, what we lack is enough evidence of effective solutions, and the focus on supervisor support is only a small part of the answer. A promising conceptual framework for a more in-depth comprehension of these mechanisms is hierarchical social support. This plays a significant role in individual functioning and well-being in the workplace, influencing the intention to leave the organization and psychological distress within the organizational context, particularly among those who perceive themselves as targets of workplace bullying.

Based on the aforementioned studies, our primary objective is to assess the associations of workplace bullying as an instigator, along with turnover intention and psychological distress as outcomes. A secondary objective is to examine the role of supervisor’s perceived support as a mediator between workplace bullying and turnover intention on one hand, and between workplace bullying and psychological distress on the other. A number of studies indicate that leadership style has an impact on the emergence of bullying [12], but other studies show the crucial role of the supervisor [7,9]. Indeed, supervisor support can act as an interface between bullying and serious consequences, such as burnout and turnover [24].

By examining supervisor support as a possible mediator between workplace bullying, the predictor, and distress and turnover intentions, the criterion measures, our contributions to the literature are threefold. First, the literature is rather scarce concerning the relationships between the four variables, even if these relationships between the variables two by two were studied. Second, with few exceptions, the mediating effects of supervisor support and their simultaneous links to distress and turnover intention have not been studied much with regard to certain sectors, with the care sector being the most explored compared to the commercial and industrial sectors. Third, many studies on distress do not examine, in the same study, the turnover intentions and, specifically, if the causes of distress and turnover are the same. This question is important because of the practical implications in different sectors.

### 1.1. Workplace Bullying and Psychological Distress

Workplace bullying is a form of aggression with negative repercussions, and it is able to produce individual psychological health problems [29,30], leading to deteriorated mental and physical health in bullied individuals [7,31,32,33,34]. Workplace bullying adversely impacts individuals’ physical and psychological health, specifically psychological distress [30,35,36,37,38]. Psychological distress is defined as “a non-specific syndrome including symptoms related to depression, anxiety, irritability, exhaustion, social disengagement, and cognitive problems” [39]. Workplace bullying is correlated with such psychological distress symptoms as anxiety, depression, and irritability [40]. For example, a longitudinal study revealed a link between workplace bullying and depression [41]. Specifically, studies have shown that workplace bullying has psychological consequences for the victim, such as headaches, sleep disorders, heart palpitations, anxiety, depression, and feelings of isolation [42,43,44]. In the same vein, being “directly exposed to higher levels of bullying acts in the workplace is associated with higher levels of psychological distress and increased turnover intentions (i.e., thinking about leaving job, looking for another job)” [45]. However, scholars [43,44] report that post-traumatic stress disorder (PTSD) symptoms appear in many bullied workers. Finally, [45] notes that being exposed to bullying can have dire consequences for mental and physical health, including depression, helplessness, anxiety, and hopelessness. The aforementioned research forms the basis for the following hypothesis:

**Hypothesis** **1.** *Workplace bullying is positively correlated to psychological distress.*

### 1.2. People Who Experience Workplace Bullying Are More Likely to Think about Leaving Their Jobs

Workplace bullying can generate responses of varying natures in individuals, with the turnover intention being one such possible reaction, which manifests itself on the behavioral level [12,18,19,20,21,22,23,24,25,46,47]. The intention to leave is “the (subjective) probability that an individual will change jobs within a certain time period” [48]. The turnover intention is to be distinguished from turnover in that a large number of studies show that the turnover intention is a predictor of turnover [49]. The turnover intention is the first step in the turnover process that leads a worker to leave their organization [50]. Several studies have highlighted the relationship between workplace bullying and the intention to leave one’s job [47,51,52]. Some authors suggest that targets’ response strategies change over time [53]. Targets appear to use conflict-resolution strategies early in the process, then change strategies several times and end up trying to leave the company. Another study showed that employees exposed to bullying had the intention to leave the company [24]. In a meta-analysis, [42] points out a positive correlation between bullying and turnover intention. More recently, exposure to psychological harassment has been shown to have a direct impact on the bullied’s turnover intention [47,54]. These studies lead us to put forward the following hypothesis.

**Hypothesis** **2.** *Workplace bullying is positively correlated to turnover intention.*

### 1.3. Workplace Bullying, Social Support at the Workplace, Turnover Intention, and Psychological Distress

Social support is characterized by “helpful social interactions with colleagues and supervisors” [55]. Regarding supervisor support, it is defined by employees’ general opinions on the recognition of their contributions and the expression of interest by supervisors in their well-being [56]. Numerous studies have underscored the crucial importance of social support as a buffer against stress [55] and in the prevention of professional burnout [57]. According to the model proposed by [55], which integrates decision latitude in the context of occupational stress, supervisor support is considered a crucial resource. However, the lack of supervisor support can create an environment conducive to the development of negative effects impacting the health and well-being of employees. Stress and professional burnout are part of psychosocial risks, and support from colleagues and the hierarchy can act as a buffer against these two risks.

Workplace bullying is increasingly acknowledged. Yet, it has long been an enduring issue within organizations, exerting negative repercussions on the physical and psychological well-being of employees, as well as on the effective functioning of organizations. By analogy, it can be concluded that support can also act as a buffer against workplace bullying. The relationship between social support and workplace bullying has been firmly established in several studies [52,58,59,60,61]. An empirical study conducted by [62] revealed a negative correlation between supervisor support and workplace bullying, a result confirmed by a recent study [63]. Employees who feel supported by their supervisors appear to be more resilient in the face of workplace bullying, while the lack of support can create an environment conducive to the proliferation of bullying behaviors.

Previous studies [64,65] have demonstrated that the actions and behaviors of supervisors impact the well-being of workers and are effective in reducing the intention to leave the organization. Supervisors, as representatives of the organization, play a crucial role in supporting their subordinates in achieving organizational goals [66]. Insufficient supervisor support can lead to issues, such as absenteeism, dissatisfaction, and a high intention to leave the company, contributing to poor performance [52]. In the same vein, [67] and [68] confirm the buffering effect of supervisor support on the organization, leading to a reduced intention to leave the company. The above-cited studies have laid the foundations, enabling us to formulate the following hypotheses.

**Hypothesis** **3.** *Perceived supervisor support is negatively correlated with workplace bullying.*

**Hypothesis** **4.** *Perceived supervisor support is negatively correlated to turnover intention and psychological distress.*

### 1.4. The Hierarchical Social Support as an Explanatory Mechanism

The present study focuses on the mediating role of hierarchical social support in two crucial relationships: first, between workplace bullying and the intention to leave the organization, and second, between workplace bullying and psychological distress. Hierarchical social support, defined as professional support within the workplace context [69,70], is traditionally recognized as a critical element in alleviating stress [71,72] and burnout [73,74,75,76,77] and promoting work engagement [72,78]. According to [79], social support is thought to affect health in three ways: (a) by regulating thoughts, feelings, and behaviors so as to promote health; (b) by fostering an individual’s sense of meaning in life; and (c) by facilitating health-promoting behaviors (e.g., diet, exercise, proper sleep, appropriate use of drugs, alcohol, and cigarettes).

Similarly, hierarchical social support can play a crucial role in reducing workplace bullying behaviors [62,69,80,81]. Studies, such as the one conducted by [82], have demonstrated that support from supervisors contributes to mitigating psychological tension among workers who have experienced workplace bullying across various sectors. Providing authentic support to individuals facing harassment is imperative, as it can contribute to restoring their confidence and promoting both physical and mental well-being [2,83].

Specifically, a less stressful work environment can decrease the risk of developing workplace bullying behaviors. However, a work environment lacking adequate support from supervisors sends a signal to employees, suggesting that the organization cannot provide appropriate, healthy, and secure working conditions [84]. Studies by [85] emphasize that social support from the hierarchy is associated with a reduction in workplace stress. Thus, a less stressful work environment may be less conducive to the development of workplace bullying behaviors.

Finally, the mentioned research converges on the fundamental idea that hierarchical social support is a crucial element in preventing workplace bullying. It fosters a healthy organizational climate and open communication and acts as an essential protective factor for employees’ psychological well-being. The combined above-cited works have laid the foundations that enable us to formulate the following hypotheses.

**Hypothesis** **5.** *Perceived supervisor support is a mediating variable in the relationship between workplace bullying and turnover intention.*

**Hypothesis** **6.** *Perceived supervisor support is a mediating variable between workplace bullying and psychological distress.*

## 2. Method

### 2.1. Procedure

The data correspond to a convenience sample. They were collected using an online questionnaire, sent either directly to contacts or via the LinkedIn professional network. We contacted around 1500 employees, and participation was open and voluntary. The general purpose of the study (e.g., to investigate interpersonal relationships at work) and the time required to complete the questionnaire (approximately 20 min) were presented to individuals at the start of the questionnaire. The participants completed an online questionnaire containing a consent form. The participants were also assured that all their answers would remain confidential and anonymous. For this data collection, the participants were sent a letter explaining the purpose of the study (i.e., to expose psychosocial risks) and inviting them to complete a paper version of the questionnaire. The letter explained that participants were asked not to give their names or other personal details in their answers, in order to preserve their confidentiality and anonymity, and that their participation was voluntary.

### 2.2. Participants

This convenience sample involved 424 workers from 70 French companies and institutions. The population is made up of 252 women (59.43%) and 172 men (40.57%) aged 18 to 65 (*M* = 38.25, *SD* = 11.25). Of the respondents, 87.8% had over 10 years of seniority in the company, and 12.2% had under 10 years. And, 71.46% were in a couple, while 28.54% were single. The number of dependent children was between 0 and 6 (*M* = 1, *SD* = 1.05). Ninety-five percent of those surveyed worked during the day and 5% did not provide information on this question. The following sectors of activity were represented: private 70% (38% females; 32% males), public 28% (20.53% females; 7.47% males), parapublic 2% (1.77% females; 0.23 males) Finally, 33 trades were represented in this study, with commercial 21% (11.92% females; 9.08% males), educational 12% (7.12% males; 4.88% females), medical 15.8% (10.19% females; 5.61% males), and industry 11.57% (10.03% males; 1.54% females) were the most prominently represented.

### 2.3. Measures

All questionnaires were administered in French. The questionnaire was presented as a survey on interpersonal relationships at work.

Leymann Inventory of Psychological Terrorization (LIPT) was a scale that consists of 45 items (Cronbach’s α = 0.93) associated with a 4-point Likert-type scale ranging from 1 (never) to 4 (once a week). The items were divided into five dimensions: self-expression effects (e.g., “Your superiors do not let you express yourself or say what you have to say”); occupational situation affects quality of life (e.g., they force you to perform absurd or useless tasks); self-contacts effects (e.g., you cannot talk to anyone, everyone avoids you); social reputation effects (e.g., they circulate false or unfounded rumors about you); and health effects (e.g., they make you do harmful or dangerous work).

To assess workplace bullying, there are two types of methods (subjective and objective). In this study, we employed the so-called objective one. This method assesses exposure to bullying by using a list of actions, where the respondents indicate if they are the targets of each action. The method focuses on describing experienced behaviors and does not directly ask if they feel bullied. This approach is widely accepted in the scientific community (e.g., [21,32,41]).

The psychological distress scale [86] consisted of 23 items (Cronbach’s α = 0.94) with 5 response possibilities on a Likert scale: from 1 (never) to 5 (almost always). This tool utilizes 23 items, with 9 measuring anxiety and depression (e.g., I feel bad about myself), 7 measuring work disengagement (e.g., I feel like giving up, quitting everything), and 7 measuring irritability and aggression (e.g., I am aggressive for no reason). The order of these items is randomized. A global score has been calculated based on the 23 items comprising the questionnaire. Studies suggest considering this global score to assess psychological distress [39,86].

The Van Veldhoven and Meijman’s turnover intention scale [87] was composed of 4 items translated into French and used in a study [88]. It consisted of four items (e.g., I sometimes consider looking for work outside this organization) associated with a 4-point Likert scale ranging from 1 (strongly disagree) to 4 (strongly agree). Cronbach’s alpha coefficient in this study was (α = 0.78).

The perceived supervisor support [89] scale included a Likert scale ranging from 1 (strongly disagree) to 4 (strongly agree) with 4 items (Cronbach’s α = 0.78, e.g., my immediate superior makes it easier to perform work).

### 2.4. Analysis

First, we checked whether our data followed a normal distribution using the Shapiro–Wilk test. The results showed that the data respected this distribution. Next, we performed analyses of variance (ANOVA) to assess the differences between the socio-demographic variables on the different dependent variables (intention to change jobs and psychological distress). In other words, intention to change jobs or psychological distress does not differ between those who have worked 10 years or more and those who have worked less than 10 years, nor between men and women, nor between different sectors, nor according to marital status. Second, in our study, we carried out a factorial analysis of LIPT in order to assess the presence of the five-factor structure. However, the results of our analysis do not confirm this expected structure. We identified four factors instead of five. These factors were characterized as follows: ‘Preventing the victim from expressing him/herself’, ‘Discrediting the victim in his/her work’, ‘Compromising the victim’s health’, and ‘Isolating the victim’. Our findings did not reveal distinct correlations between different aspects of bullying and the studied variables. This suggests consistency in how these bullying behaviors impact individuals’ psychological responses and attitudes in the workplace.

## 3. Results

### 3.1. Correlationnal Analyses

For the correlations (Table 1), we did not perform any specific calculations to determine the effect size. We examined the Pearson correlation coefficient and interpreted it according to Cohen’s benchmarks (1988).

Correlational analyses (Table 1) show that workplace bullying is negatively correlated with support from a supervisor (*r* = −0.54; *p* < 0.05, this correlation is considered medium according to Cohen) and positively with the intention to leave (*r* = 0.30; *p* < 0.05, this correlation is considered weak according to Cohen) and psychological distress (*r* = 0.51; *p* < 0.05, this correlation is considered medium according to Cohen). In addition, support from a supervisor is negatively correlated with the intention to leave (*r* = −0.39; *p* < 0.05, this correlation is considered weak according to Cohen) and psychological distress (*r* = −0.40; *p* < 0.05, this correlation is considered weak according to Cohen). Intention to leave is positively correlated with psychological distress (*r* = 0.35; *p* < 0.05, this correlation is considered weak according to Cohen).

### 3.2. Mediations Analysis

**Figure 1:**
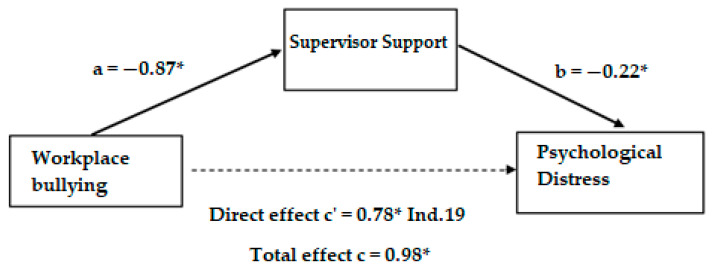
Mediation results with workplace bullying (IV) and psychological distress as the dependent variable. * p < 0.05.

For testing the mediation hypotheses, we used [90] “SPSS 28.0” macro. The advocated approach [89], based on regressions, calculates the mediation effect or indirect effect as the product of link A (specific effect of an IV on a mediating variable abbreviated as MV) and link B (specific effect of an MV on the DV). Link C represents the total effect of an IV on the DV. The bootstrapping method is used in this study to verify the mediating role played by social support from a supervisor in the relations between workplace bullying and psychological distress on the one hand and between workplace bullying and intention to leave on the other hand. Thus, for our first mediation (Figure 1), workplace bullying (*ß* = 0.78, *p* < 0.001) explains psychological distress (link C′) and contributes negatively (*ß* = −0.87, *p* < 0.001) to supervisor support (Link A). Social support from a supervisor (*ß* = −0.22, *p* < 0.001) negatively influences psychological distress (link B), and ultimately, by removing the indirect effect (*ß* = 0.19) from the total effect (link C), workplace bullying (*ß* = 0.98, *p* < 0.001) still explains psychological distress with a confidence interval of [−0.29; −0.15]. Therefore, these results lead us to conclude that supervisor support effectively functions as a mediator in the relationship between workplace bullying and psychological distress. This mediation is partial, as by keeping the mediating variable (supervisor support), the link between workplace bullying and psychological distress remains positive.

**Figure 2:**
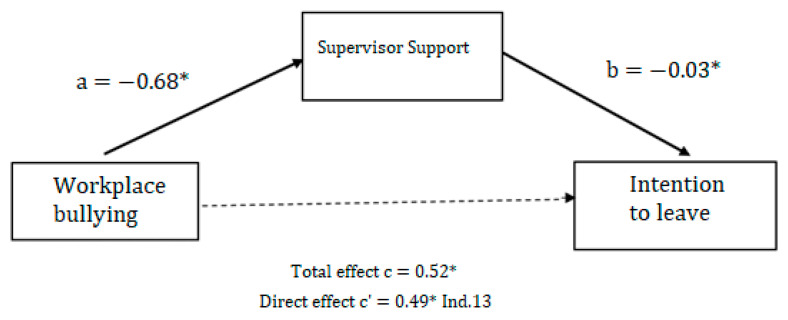
Mediation results with workplace bullying (IV) and, intention to leave as teg dependent variable. * p < 0.05.

Regarding the second mediation (Figure 2), workplace bullying (*ß* = 0.52, *p* < 0.05) explains the intention to leave (link C) and negatively (*ß* = −0.68, *p* < 0.05) contributes to support from a supervisor (Link A). Social support from a supervisor (*ß* = −0.03, *p* < 0.05) negatively influences psychological distress (link B), and ultimately, by removing the indirect effect (*ß* = 0.13) from the total effect (link C), workplace bullying (*ß* = 0.49, *p* < 0.05) still explains the intent to quit with a confidence interval of [0.07; 0.27]. This second relationship once again confirms the mediating role of hierarchical support in the relationship between workplace bullying and turnover intention, even though this mediation may be partial according to the statistical results above.

These two results validate our Hypotheses 5 and 6.

## 4. Discussion

The objective of this study was twofold: first, to examine the associations between workplace harassment, psychological distress, and the intention to leave the organization; and second, to analyze the direct and indirect effects (via perceived supervisor support) of workplace harassment on psychological distress and the intention to leave the organization among French employees. The results of this study are useful in several ways. First, workplace bullying is positively correlated with psychological distress, confirming our first hypothesis. Specifically, this positive correlation highlights a significant relationship, suggesting that individuals experiencing workplace bullying are more likely to exhibit increased levels of psychological distress. Consequently, this psychological distress can exert a detrimental influence on employees’ psychological well-being. On an individual level, employees subjected to workplace bullying may develop mental health issues, such as anxiety, depression, or even post-traumatic symptoms. These problems can not only affect work performance but also have repercussions on individuals’ personal lives. This result aligns with the previous research that establishes a consistent link between workplace bullying and adverse effects on mental health [7,29,31,32,33,34,36].

Second, a positive link was found between workplace bullying and the intention to leave the organization, confirming our second hypothesis. This positive correlation can be explained by several factors. For instance, experiencing workplace bullying can create a toxic work environment that significantly impacts employees’ psychological well-being. The resulting anxiety, depression, and disengagement can lead to decreased job satisfaction and an increased desire to leave the organization. Additionally, workplace bullying can compromise the quality of professional relationships, fostering feelings of isolation and a lack of social support within the organization. Employees facing such situations may perceive leaving the company as a strategy to escape a detrimental work environment. This positive link is consistent with the prior research that establishes a connection between workplace bullying and negative psychological symptoms, such as depression, anxiety, and hopelessness [4,46,47]. Recent studies, such as that by [5], show that employees exposed to workplace bullying report significantly higher intentions to leave the organization.

Furthermore, in line with our third and fourth hypotheses, we observed a negative relationship between hierarchical social support, workplace bullying, intention to leave the organization, and psychological distress. In other words, this suggests that a work environment where employees feel supported by their hierarchical superiors is less likely to tolerate workplace bullying. Employees in such environments are less likely to experience psychological distress and are not inclined to leave their organization. For example, in an organization where hierarchy encourages open communication, respect, and constructive conflict resolution, employees are less likely to be targets of workplace bullying. Conversely, in an organization where the hierarchy does not take measures to promote a respectful work environment and does not respond effectively to reports of bullying, employees may feel isolated and vulnerable. The lack of support may also encourage employees to leave the organization and experience psychological distress for various reasons (e.g., stress, conflicts among colleagues, workplace bullying, and injustice) due to a lack of confidence that their complaints will be taken seriously. This result is consistent with the literature [52,58,59,60,61,62]. Recent studies, such as that by [63], confirm this negative link between hierarchical social support and workplace bullying. Employees who feel supported by their superiors may be more resilient to workplace bullying, while a lack of support can create an environment conducive to the proliferation of bullying behaviors, psychological distress, and the intention to leave the organization. These examples and cited studies illustrate how the level of hierarchical social support can influence employee engagement and psychological well-being. Organizations can learn from these findings by implementing initiatives to strengthen social support, such as training programs for managers, open communication channels, and regular feedback mechanisms to address employees’ psychological and professional needs.

Finally, our findings confirm the mediating role of hierarchical social support, partially validating our fifth and sixth hypotheses. Specifically, hierarchical social support is a mediator variable in both the relationships between workplace bullying and the intention to leave the organization and between workplace bullying and psychological distress. These findings are groundbreaking, as they support the conclusions of other studies on workplace bullying. This study tests the mediating role of supervisory support in the relationship between workplace bullying and psychological distress on one hand and between workplace bullying and the intention to leave the organization on the other. By highlighting the mediating role of hierarchical social support, our results suggest that this type of support for employees can act as a barrier against the devastating consequences of workplace bullying. For example, in organizational contexts that promote empathetic leadership and open communication channels, employees are likely to feel supported, limiting psychological distress and the desire to leave the organization. These findings are in line with previous studies showing that hierarchical social support can play a mediating role, particularly in mitigating stress [71,72] and burnout [73,77] and promoting work engagement [72,78], playing a crucial role in reducing psychological distress and the intention to leave one’s job in the face of workplace bullying [62,69,80,81]. For instance, [83] demonstrated that the support of supervisors and colleagues contributes to alleviating the psychological tension among workers who have experienced workplace bullying across various sectors. Some recent studies have confirmed this link in the context of workplace bullying [52,62,90] and in cyber workplace bullying [44]. For example, [91] showed, in a study conducted among nurses, that workplace bullying was negatively related to job satisfaction, sleep problems, the need for recovery, and emotional exhaustion through relaxation, but this is moderated among nurses who perceive a high level of support from their supervisor. These results demonstrate that employees facing workplace bullying must benefit from hierarchical social support, as it can contribute to restoring their confidence and promoting their physical and mental well-being. Specifically, our results offer a significant contribution to the literature by empirically substantiating the mechanisms of hierarchical social support in the complex relationship between workplace bullying and the repercussions on employees’ psychological well-being and organizational attachment.

In other words, psychological aid from the supervisor would allow the individual to make a more favorable assessment of the situation and demands at work, with the feeling of being supported, thus reducing the psychological distress and turnover intention. In addition, the victim would face the acts that the bully subjects him to (isolation, slander, criticism, humiliation, and mental and physical aggression) with a sense of less vulnerability and isolation [31]. With social support allowing a person to reach out for support in case of difficulties, said person feels they belong to a social group and does not feel alone.

### Limitations and Perspective Applications

This study has limitations that bear mentioning. The first limitation concerns the sample. The sample may suffer from a lack of homogeneity due to a smaller proportion of men than women. It would be interesting to redo this study with a larger number of male employees to test the similarity or not of the results between men and women. In addition, despite the large sample size (424 participants), this was a convenience sample. As a result, employees were drawn from 70 companies (on average, six people from each business). Data were collected using an online questionnaire, which was sent either directly to contacts or through the LinkedIn professional network. As an online professional network, LinkedIn is mainly used for professional branding. Employees participate as members of their company, but mainly as their own marketing agent looking for the best professional opportunities. Therefore, our sample was not targeted at companies but at professionals working in different organizations. Thus, our study was confronted with these problems and limitations. Future studies need to target organizations directly in order to achieve a better representation of the employees in each sector. Second, the use of a cross-sectional methodology does not make it possible to establish the causal relationships between the variables. It would, therefore, be appropriate, in future research, to carry out longitudinal studies in order to provide additional information about the meaning of the relationships between variables. Common variance bias may have influenced the results by increasing the strength of the correlations, since all data were collected using the same method [92]. Third, regarding the measuring instrument used, the responses were self-reported and may have been influenced by some biases (halo). Qualitative and longitudinal studies could usefully supplement this study to better understand the specific links between different forms of social support (from colleagues, external and perceived organizational support, family, friends, etc.). Finally, [89] is a little outdated, but we wanted to choose a scale that measures both coworker’s relationships and supervisor support. Finally, for POS, this is a more abstract organizational level that involves taking political consciousness, measures, and feelings about how employees are treated and accompanied in a general way but not necessarily adapted to the particular needs of the person.

## 5. Implications for Practical Applications

In a workplace context characterized by increasingly frequent and drastic changes, supervisor social support stands out as one of the key factors in safeguarding individuals experiencing work-related challenges. This form of social support also fosters a more positive work climate, given its significant impact on strengthening interpersonal relationships and fostering a sense of belonging, while mitigating the negative effects of workplace bullying behaviors. An individual subjected to workplace bullying who receives support from their superiors may experience reduced distress and a decreased inclination to leave the organization. Consequently, the cultivation of managerial support should be prioritized by organizations to prevent and address instances of workplace bullying.

In practical terms regarding this study, superiors can act on several fronts. First, in the domain of household awareness and training, they should be trained to recognize signs of workplace bullying and understand its impact on employees as was recommended [31,44,59]. Additionally, awareness of the importance of organizational support in reducing employees’ psychological distress is crucial [31,44,93]. Second, concerning policies and procedures, companies should develop and implement zero-tolerance policies regarding workplace bullying [36,44]. This should include clear procedures for reporting and addressing cases of bullying, while fostering an organizational support environment where employees feel supported and valued. Third, regarding executive training, it is crucial that executives and hierarchical managers are trained to provide adequate support to employees facing situations of workplace bullying [31]. This may include skills in active listening, conflict resolution, and stress management. Fourth, concerning organizational culture, companies must promote a culture that values support among team members [68,70]. By adopting these measures, households can contribute to creating a healthier and more supportive work environment that is beneficial for both employee retention and psychological well-being.

## Figures and Tables

**Table 1:** Means, standard deviations, Cronbach’s alpha, and correlations between workplace bullying, turnover intention, psychological distress, and supervisor support.

Scale	*M*	*SD*	1	2	3	4
1. Workplace bullying	1.44/4	0.47	**0.93**			
2. Supervisor support	2.72/4	0.63	−0.54 *	**0.78**		
3. Turnover Intention	2.41/4	0.80	0.30 *	−0.39 *	**0.85**	
4. Psychological distress	2.79/5	0.51	0.51 *	−0.40 *	0.35 *	**0.94**

*N* = 424; *M* = mean; *SD* = standard deviation; bolded Cronbach’s Alpha on the diagonal, * *p* < 0.05.

## Data Availability

The original contributions presented in the study are included in the article, further inquiries can be directed to the corresponding author.
